# Design and Modeling of a Curved Beam Compliant Mechanism with Six Degrees of Freedom

**DOI:** 10.3390/mi13020208

**Published:** 2022-01-28

**Authors:** Sheng Lin, Jiacheng Wang, Wenkang Xiong, Qingyuan Hu, Hui Liu, Qi Wang

**Affiliations:** 1 School of Mechanical Engineering, Dalian Jiaotong University, Dalian 116028, China; wangjiacheng9803@163.com (J.W.); xiognwenkang@163.com (W.X.); 18954068469@163.com (H.L.); wangqi1998123@163.com (Q.W.); 2 School of Science, Jiangnan University, Wuxi 214122, China; qingyuanhu@jiangnan.edu.cn

**Keywords:** compliant mechanism, curved beam, isogeometric analysis

## Abstract

Compliant mechanisms are widely used in cutting-edge scientific and technological fields such as precision engineering, micro-/nano-manipulation, or microelectronics. Hence, the demand for multi-degree-of-freedom compliant mechanisms has increased sharply. The structure of compliant mechanisms becomes increasingly complex with the increase of degrees of freedom. Here, a compliant mechanism with six degrees of freedom is proposed based on curved beams. The compliant mechanism has the advantages of simple structure and multi-degree-of-freedom. Using the isogeometric analysis method, a model of the mechanism is constructed. Static analysis show that six degrees of freedom can be generated. The prototype of the mechanism is developed by 3D printing. A loading test in six degrees of freedom is carried out. The output and input have high linear relations and the structure has low inter-directional coupling. We trust that this study provides a pioneering step towards the design of compliant mechanisms based on curved beam elements.

## 1. Introduction

Due to advantages such as no requirement for lubrication, no backlash, and less assembly [1,2,3,4,5], compliant mechanisms are widely used in the fields of precision processing, biological cell manipulation, microelectronics and micro-/nano-manipulation [6,7,8,9,10]. The demand of multi degrees of freedom leads to the structure of compliant mechanism becoming increasingly complex. Complex configurations in compliant mechanisms usually lead to intractable designing and manufacturing [11,12]. Hence, developing a kind of compliant mechanism with a simple structure and multi degrees of freedom is urgent to meet growing demand for multi degrees of freedom compliant mechanisms.

Multi-degree-of-freedom can always be realized through the parallel, serial, or hybrid flexible hinges or compliant straight beams [13]. The series compliant mechanism can provide more degrees of freedom, but series compliant mechanisms are rarely used because of their low stiffness. Parallel and hybrid approaches are common configurations of compliant mechanisms. Hopkins [14,15] presented the freedom and constraint topology method and a variety of multi degrees of freedom complex compliant mechanisms have been designed. Some hybrid compliant mechanisms based on the straight beams were synthesized with this method. Nelson [16] employed folding techniques from origami to develop a hybrid compliant mechanism with multi degrees of freedom. The complex compliant mechanism is difficult to made. Pinskier [17] investigated a four degrees of freedom parallel-serial slave mechanism. a methodology was presented to precisely control the motion of a multi degrees of freedom piezo-actuated flexure mechanism with haptic feedback. Zheng [18] proposed a hybrid compliant mechanism with a flexible central chain and a cantilever beam. The relationship between the moving platform and parallel kinematic chains was derived. Jiang [19] presented a compact flexure-based decoupler with two degrees of freedom. The decoupler is a compliant mechanism with parallel configuration. Yu [20] applied freedom and constraint topology to design a compliant parallel mechanism with two rotational degrees of freedom, which meets the requirement of a lightweight and compact pan-tilt platform. Ruiz [21] proposed a three-PRS compliant parallel manipulator of three degrees of freedom. The solid body kinematics model of the three-PRS was established. Zhu [22] synthesized a six degrees of freedom spatial compliant mechanism combined the topology optimization method with the isomorphic mapping matrix. The geometry after topology optimization was not standardized, and the establishment of the model was relatively difficult. Yu [23] designed a three prismatic-prismatic-revolute planar compliant parallel mechanism with three degrees of freedom. The mechanical structure guaranteed the motions along/about the specific axes to improve motion accuracy. Telleria [24] proposed design rules and models for the synthesis and optimization of cylindrical flexures. These new flexures systems could meet the requirement for applications constrained to a cylindrical geometry. However, the flexible element is only limited to circular curved beams. It is difficult to model arbitrary curved beams. As mentioned above, the simple structure and multi degrees of freedom always conflict with each other for the compliant mechanism based on flexible hinges or compliant straight beams. A curved beam compliant mechanism has a certain contribution to solve this contradiction, but the existing curved beam compliant mechanism is only limited to cylindrical geometry, and the modeling method is only limited to a circular arc curved beam.

This research presents the two main contributions to solve the contradiction of simple structure and multi degrees of freedom. (i) Taking advantage of a curved beam with multi degrees of freedom, a six degrees of freedom spatial compliant mechanism based on the curved beam is proposed. (ii) Isogeometric analysis is utilized to model the compliant mechanism based on curved beam elements. Application of isogeometric analysis solves the problem of modeling the compliant mechanism based on a curved beam with an arbitrary shape.

## 2. Design of the Compliant Mechanism Based on Curved Beam Compliant Element

**Figure 1:**
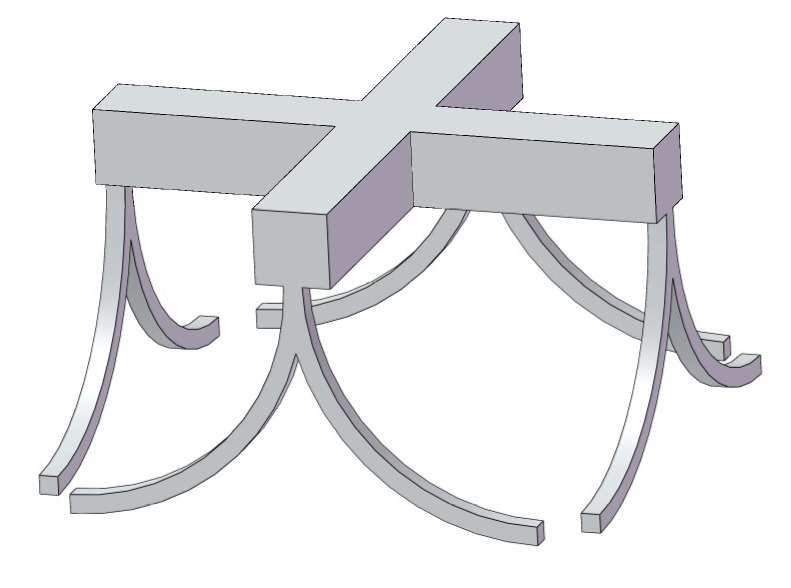
Prototype of the six degrees of freedom compliant mechanism.

A six degrees of freedom compliant mechanism with simple structure is designed based on a curved beam compliant element. The compliant mechanism is designed by combining eight compliant curved beams and a worktable, as shown in Figure 1.

**Figure 2:**
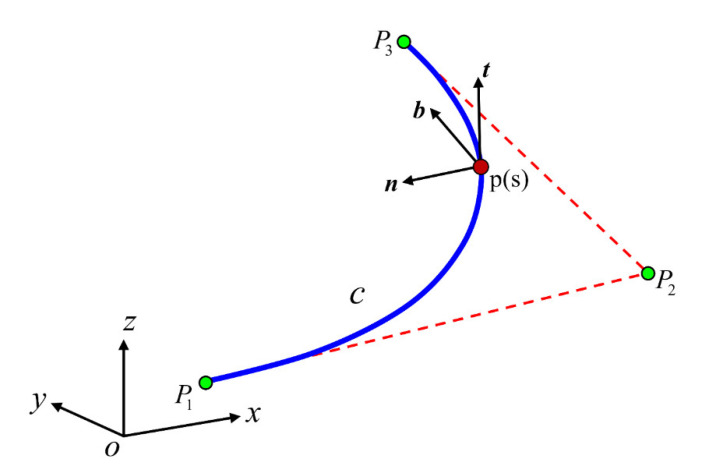
A spatial curved beam and its Frenet frame at a given point p(s).

Non-uniform rational B-splines (NURBS) *c*(*x*, *y*, *z*) is employed to construct the curved beam model [25], which is shown in Figure 2. The Frenet frame (***t***, ***n***, ***b***) is used to define the local coordinate system. $t(t_{x},t_{y},t_{z})$ is the unit tangent vector, $n(n_{x},n_{y},n_{z})$ is the unit normal vector and $b(b_{x},b_{y},b_{z})$ is the unit binormal vector.

The parameter equation of the curved beam is defined by [26]

(1) $$\left\{\begin{array}{c} x(\xi )=\sum_{i=1}^{n}R_{i}(\xi )x_{i}\\ y(\xi )=\sum_{i=1}^{n}R_{i}(\xi )y_{i}\\ z(\xi )=\sum_{i=1}^{n}R_{i}(\xi )z_{i} \end{array}\right.$$

where, $P_{i}=(x_{i},y_{i},z_{i})$ is the *i*-th NURBS control point, $R_{i}=\frac{N_{i,p}\left(\xi \right)\omega_{i}}{\sum_{j=1}^{n}N_{i,p}\left(\xi \right)\omega_{j}}$ are the basis function of the NURBS, n is the number of control points, $\omega_{i}$ represents the *i*-th weight, ξ is the parameter coordinate.

$N_{i,p}(\xi )$ is the *i*-th B-spline basis function with order *p*, and the definition of $N_{i,p}(\xi )$ is:

(2) $$N_{i,0}(\xi )\left\{\begin{array}{l} 1,\;if\;\xi_{i}\leq \xi \leq \xi_{i+1}\\ 0,\;otherwise \end{array}\right.$$

(3) $$N_{i,p}(\xi )=\frac{\xi -\xi_{1}}{\xi_{i+p}-\xi_{i}}N_{i,p-1}(\xi )+\frac{\xi_{i+p+1}-\xi }{\xi_{i+p+1}-\xi_{i+1}}N_{i+1,p-1}(\xi )$$

**Figure 3:**
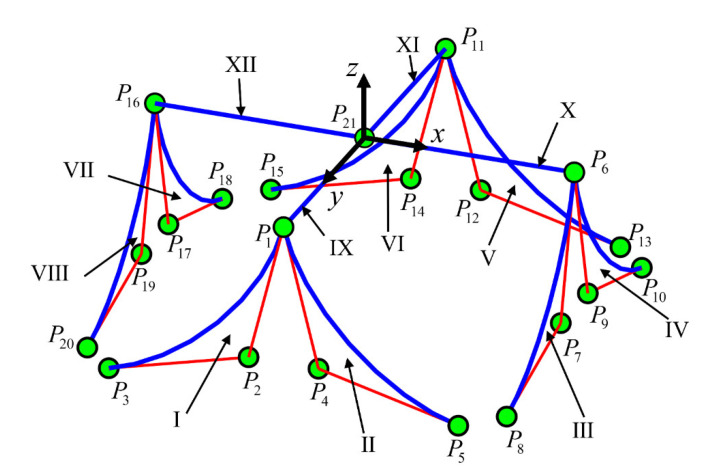
Model of spatial curved beam compliant mechanism based on non-uniform rational B-splines (NURBS).

The worktable is simplified to four rigid straight beams, and the curved beams are replaced by NURBS. The geometric model of the curved beam compliant mechanism can be simplified to Figure 3.

## 3. Modeling of the Compliant Mechanism with Isogeometric Analysis

The isogeometric analysis method is employed to establish the model of the compliant mechanism. In the geometric method, the NURBS basis function is used as the shape function of the element. Then it is convenient to obtain the relationship between stiffness matrix and geometric parameters.

As shown in Figure 2, the Frenet frame (***t***, ***n***, ***b***) is used to define the local coordinate system. The orthonormal triad can be calculated by:

(4) $$t\left(\xi \right)=\frac{\dot{c}\left(\xi \right)}{\left|\dot{c}\left(\xi \right)\right|},\;n\left(\xi \right)=\frac{\ddot{c}\left(\xi \right)}{\left|\ddot{c}\left(\xi \right)\right|}\;,\;b=t\times n$$

where, the superscript $\dot{\nu }(\xi )$ means dν/dξ, |ν| means the module length of vector ν and $\left(\nu_{1}\times \nu_{2}\right)$ means the vector product of vectors $\nu_{1}$ and $\nu_{2}$.

In the Frenet framework, the strain formula can be derived as [27]:

(5) $$\left[\begin{array}{l} \varepsilon_{t}\\ \varepsilon_{n}\\ \varepsilon_{b}\\ \gamma_{t}\\ \gamma_{n}\\ \gamma_{b} \end{array}\right]=\left[\begin{array}{c} \frac{du_{t}}{ds}-\kappa u_{n}\\ \frac{du_{n}}{ds}+\kappa u_{t}-\tau u_{b}-\theta_{b}\\ \frac{du_{b}}{ds}+\tau u_{n}+\theta_{n}\\ \frac{d\theta_{t}}{ds}-\kappa \theta_{n}\\ \frac{d\theta }{ds}+\kappa \theta_{t}-\tau \theta_{b}\\ \frac{d\theta_{b}}{ds}+\tau \theta_{n} \end{array}\right]$$

where, d*s* is the infinitesimal arch length, κ is the curvature of the curved beam, τ is the torsion of the curved beam, $\tilde{u}={\left[u_{t},u_{n},u_{b}\right]}^{T}$ and $\tilde{\theta }={\left[\theta_{t},\theta_{n},\theta_{b}\right]}^{T}$ are defined as displacement and rotation fields in local coordinates, $u={\left[u_{x},u_{y},u_{z}\right]}^{T}$ and $\theta ={\left[\theta_{x},\theta_{y},\theta_{z}\right]}^{T}$ are the displacement and rotation fields in global coordinates.

Then the local strain with respect to the global variables is derived as [28]:

(6) $$\left[\begin{array}{c} \varepsilon_{t}\\ \varepsilon_{u}\\ \varepsilon_{b}\\ \theta_{t}\\ \theta_{n}\\ \theta_{b} \end{array}\right]=\left[\begin{array}{c} t_{x}\frac{du_{x}}{ds}+t_{y}\frac{du_{y}}{ds}+t_{z}\frac{du_{z}}{ds}-\kappa \left(n_{x}u_{x}+n_{y}u_{y}+n_{z}u_{z}\right)\\ n_{x}\frac{du_{x}}{ds}+n_{y}\frac{du_{y}}{ds}+n_{z}\frac{du_{z}}{ds}-\kappa \left(t_{x}u_{x}+t_{y}u_{y}+t_{z}u_{z}\right)-b_{x}\theta_{x}-b_{y}\theta_{y}-b_{z}\theta_{z}\\ b_{x}\frac{du_{x}}{ds}+b_{y}\frac{du_{y}}{ds}+b_{z}\frac{du_{z}}{ds}+n_{x}\theta_{x}+n_{y}\theta_{y}+n_{z}\theta_{z}\\ t_{x}\frac{d\theta_{x}}{ds}+t_{y}\frac{d\theta_{y}}{ds}+t_{z}\frac{d\theta_{z}}{ds}-\kappa \left(n_{x}\theta_{x}+n_{y}\theta_{y}+n_{z}\theta_{z}\right)\\ n_{x}\frac{d\theta_{x}}{ds}+n_{y}\frac{d\theta_{y}}{ds}+n_{z}\frac{d\theta_{z}}{ds}-\kappa \left(t_{x}\theta_{x}+t_{y}\theta_{y}+t_{z}\theta_{z}\right)\\ b_{x}\frac{d\theta_{x}}{ds}+b_{y}\frac{d\theta_{y}}{ds}+b_{z}\frac{d\theta_{z}}{ds} \end{array}\right]$$

In isogeometric analysis, both geometry and solution space are obtained on the basis of NURBS. Therefore, the displacements and rotations in the global coordinate system are established as:

(7) $$\begin{array}{l} u_{x}\left(\xi \right)=\sum_{i=1}^{n}R_{i}\left(\xi \right)u_{xi},\;u_{y}\left(\xi \right)=\sum_{i=1}^{n}R_{i}\left(\xi \right)u_{yi},\;u_{z}\left(\xi \right)=\sum_{i=1}^{n}R_{i}\left(\xi \right)u_{zi}\\ \theta_{x}\left(\xi \right)=\sum_{i=1}^{n}R_{i}\left(\xi \right)\theta_{xi},\;\theta_{y}\left(\xi \right)=\sum_{i=1}^{n}R_{i}\left(\xi \right)\theta_{yi},\;\theta_{z}\left(\xi \right)=\sum_{i=1}^{n}R_{i}\left(\xi \right)\theta_{zi} \end{array}$$

According to the formula $\varepsilon =B\cdot \delta^{e}$, the strain matrix B can be expressed as:

(8) $$B=\left[\begin{array}{cccccc} t_{x}\frac{dR_{j}}{ds}-\kappa n_{x}R_{j} & t_{y}\frac{dR_{j}}{ds}-\kappa n_{y}R_{j} & t_{z}\frac{dR_{j}}{ds}-\kappa n_{z}R_{j} & 0 & 0 & 0\\ n_{x}\frac{dR_{j}}{ds}-\kappa t_{x}R_{j} & n_{y}\frac{dR_{j}}{ds}-\kappa t_{y}R_{j} & n_{z}\frac{dR_{j}}{ds}-\kappa t_{z}R_{j} & -b_{x}R_{j} & -b_{y}R_{j} & -b_{z}R_{j}\\ b_{x}R_{j} & b_{y}R_{j} & b_{z}R_{j} & n_{x}R_{j} & n_{y}R_{j} & n_{z}R_{j}\\ 0 & 0 & 0 & t_{x}\frac{dR_{j}}{ds}-\kappa n_{x}R_{j} & t_{y}\frac{dR_{j}}{ds}-\kappa n_{y}R_{j} & t_{z}\frac{dR_{j}}{ds}-\kappa n_{z}R_{j}\\ 0 & 0 & 0 & n_{x}\frac{dR_{j}}{ds}+\kappa t_{x}R_{j} & n_{y}\frac{dR_{j}}{ds}+\kappa t_{y}R_{j} & n_{z}\frac{dR_{j}}{ds}+\kappa t_{z}R_{j}\\ 0 & 0 & 0 & b_{x}R_{j} & b_{y}R_{j} & b_{z}R_{j} \end{array}\right]$$

For linear equilibrium, the constitutive matrix is:

(9) $$D=\mathrm{digag}\left(EA,k_{n}GA,k_{b}GA,GI_{t},EI_{n},EI_{b}\right)$$

where, *E* and *G* are the Young and shear modulus, *A* is the area of the section of the beam, $I_{t}$ is the inertia of torsion, $I_{n}$ and $I_{b}$ are the moment of inertia of the cross section, $k_{n}$ and $k_{b}$ are the shear correction factors for the local *n* and *b* axes, respectively.

According to isoparametric transformation [29,30], the relation between infinitesimal arch length s and the parametric coordinate ξ is defined as:

(10) ds=J(ξ)dξ

and

(11) $$J(\xi )=\sqrt{{\left(\dot{x}(\xi )\right)}^{2}+{\left(\dot{y}(\xi )\right)}^{2}+{\left(\dot{z}(\xi )\right)}^{2}}$$

A non-zero knot vector interval is regarded as an element, and the element stiffness matrix in an element $\left[\xi_{i},\xi_{i+1}\right]$ is calculated by:

(12) $$K^{e}=\int_{\xi_{i}}^{\xi_{i+1}}B^{T}DBJd\xi$$

As shown in Figure 3, each curved beam has three control points, and each control point has six degrees of freedom. Then the stiffness matrix of curved beam element can be expressed as:

(13) $$K^{e}=\overset{\begin{array}{ccc} \;i\; & j\; & \;m \end{array}}{\left[\begin{array}{ccc} K_{ii}^{e} & K_{ij}^{e} & K_{im}^{e}\\ K_{ji}^{e} & K_{jj}^{e} & K_{jm}^{e}\\ K_{mi}^{e} & K_{mj}^{e} & K_{mm}^{e} \end{array}\right]}\begin{array}{c} i\\ j\\ m \end{array}$$

where, $K_{rs}^{e}(r,s=i,j,m)$ is the 6-order submatrix, the values corresponding to i,j and m are the control point number of the curved beam element.

For example, the i,j and m of the curved beam element ΙΙ in Figure 3 are 1, 4 and 5 respectively, and the element stiffness matrix is:

(14) $$K^{e}=\overset{\begin{array}{lll} \;1\; & \;4\;\; & 5 \end{array}}{\left[\begin{array}{ccc} K_{11}^{e} & K_{14}^{e} & K_{15}^{e}\\ K_{41}^{e} & K_{44}^{e} & K_{45}^{e}\\ K_{51}^{e} & K_{54}^{e} & K_{55}^{e} \end{array}\right]}\begin{array}{c} 1\\ 4\\ 5 \end{array}$$

The worktable is replaced by four straight beam elements. The Young’s modulus of the straight beam is set to $10^{6}$ times that of the curved beam, so that the worktable can be approximately regarded as a rigid body. The straight beam element stiffness matrix is a 12-order matrix, which is expressed as:

(15) $$K^{e}=\overset{\begin{array}{ll} \;i\;\; & j \end{array}}{\left[\begin{array}{cc} K_{ii}^{e} & K_{ij}^{e}\\ K_{ji}^{e} & K_{jj}^{e} \end{array}\right]}\begin{array}{c} i\\ j \end{array}$$

where, $K_{rs}^{e}(r,s=i,j)$ is the 6-order submatrix, the values corresponding to *i* and *j* are the control point number of the straight beam matrix.

The stiffness matrices of straight beams and curved beams are all obtained in the global coordinate system. All the element stiffness matrices are assembled to the global stiffness matrix, which is as follows,

(16) $$K=\overset{\begin{array}{ccccccccc} 1\; & \cdots \; & i\; & \cdots \; & j\; & \cdots \; & m\; & \cdots \; & \;n \end{array}}{\left[\begin{array}{ccccccccc} K_{11} & \cdots & K_{1i} & \cdots & K_{1j} & \cdots & K_{1m} & \cdots & K_{1n}\\ \vdots & & \vdots & & \vdots & & \vdots & & \vdots \\ K_{i1} & \cdots & K_{ii}+K_{ii}^{e} & \cdots & K_{ij}+K_{ij}^{e} & \cdots & K_{im}+K_{im}^{e} & \cdots & K_{in}\\ \vdots & & \vdots & & \vdots & & \vdots & & \vdots \\ K_{j1} & \cdots & K_{ji}+K_{ji}^{e} & \cdots & K_{jj}+K_{jj}^{e} & \cdots & K_{jm}+K_{jm}^{e} & \cdots & K_{jn}\\ \vdots & & \vdots & & \vdots & & \vdots & & \vdots \\ K_{m1} & \cdots & K_{mi}+K_{mi}^{e} & \cdots & K_{mj}+K_{mj}^{e} & \cdots & K_{mm}+K_{mm}^{e} & \cdots & K_{mn}\\ \vdots & & \vdots & & \vdots & & \vdots & & \vdots \\ K_{n1} & \cdots & K_{ni} & \cdots & K_{nj} & \cdots & K_{nm} & \cdots & K_{nn} \end{array}\right]}\begin{array}{c} 1\\ \vdots \\ i\\ \vdots \\ j\\ \vdots \\ m\\ \vdots \\ n \end{array}$$

According to the derivation process of Formulas (4)–(16), the global stiffness matrix of the compliant mechanism can be obtained according to the geometric parameters NURBS curve and material properties of the curved beam. According to Formula (16), the input–output relationship of the compliant mechanism is:

(17) F=K⋅δ

where, K represents the global stiffness matrix, F represents the load vector and δ represents the node displacement vector.

**Figure 4:**
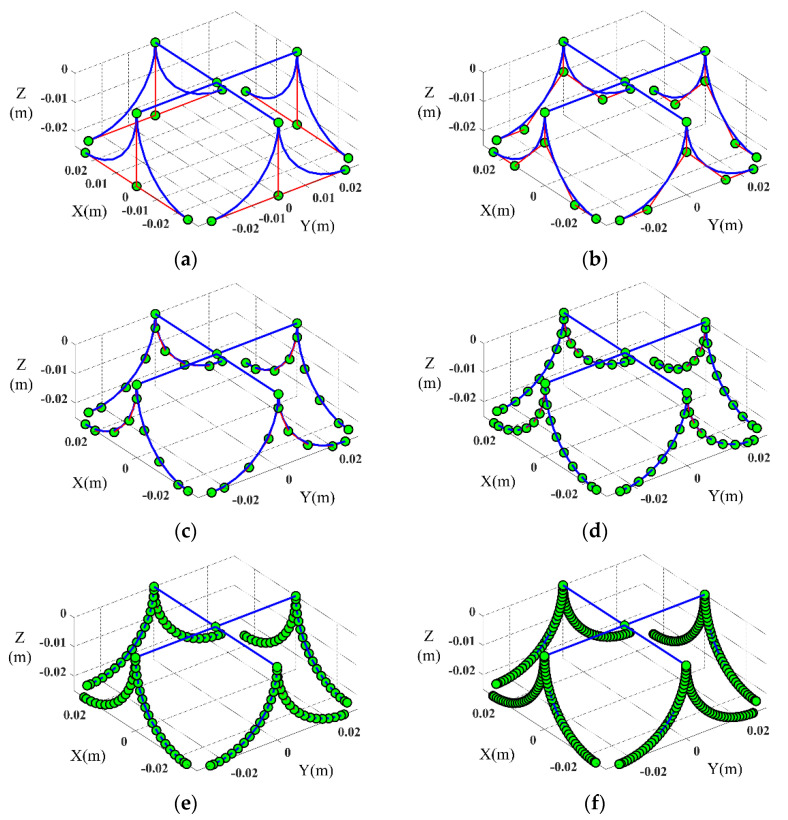
Refinement of the curved beam with different times. (**a**) The compliant mechanism without refinement. (**b**) The compliant mechanism with 1 time refinement. (**c**) The compliant mechanism with 2 times refinement. (**d**) The compliant mechanism with 3 times refinement. (**e**) The compliant mechanism with 4 times refinement. (**f**) The compliant mechanism with 5 times refinement.

Mesh refinement can improve the accuracy of isogeometric analysis. The k-refinement method is to increase the order first, then insert the node [31]. The Young’s modulus of material E is set as 1.6 GPa, Poisson ratio ν is set as 0.4 and the density ρ is set as 1.37 g/mm^3^. Figure 4 shows the mesh refinement of the mechanism by using the k-refinement method. Figure 4a is the compliant mechanism without refinement, the order p is 2, and each curved beam has three control points. Figure 4b–f are the compliant mechanism with different refinement times. With the increase of refinement times, the number of control points increases, and the control points are closer to the curve. Increasing the refinement times can effectively improve the calculation accuracy.

**Figure 5:**
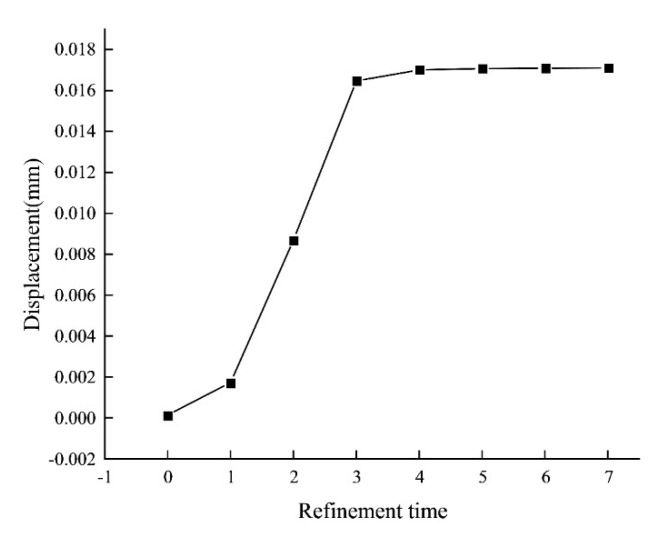
Convergence curve of displacement with refinement time.

In order to obtain reasonable subdivision times, the convergence analysis of output displacement is carried out by increasing the refinement times. Figure 5 shows the displacements of worktable center point with different refinement times. The data begin to remain stable over the five times of refinement. Therefore, the curved beam elements with five times of refinement are selected.

Apply the load to the center control point of the worktable. Under the maximum load of 120 N in each direction, the displacement along the *X*-axis is 0.0173 mm, the displacement along the *Y*-axis is 0.0170 mm, the displacement along the *Z*-axis is 0.0083 mm, the rotation around the *X*-axis is 1.064 rad, the rotation around the *Y*-axis is 1.065 rad, and the rotation around the *Z*-axis is 0.561 rad. The displacements and rotations under the maximum load of 120 N are shown in Table 1.

**Figure 6:**
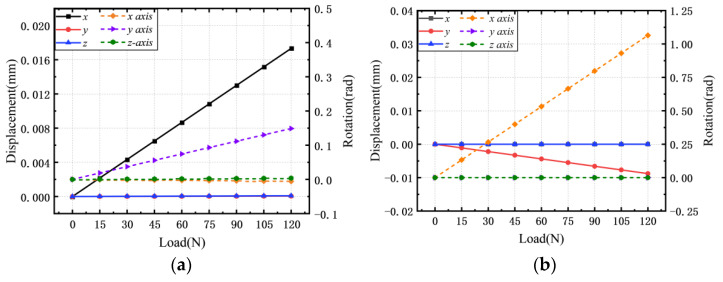

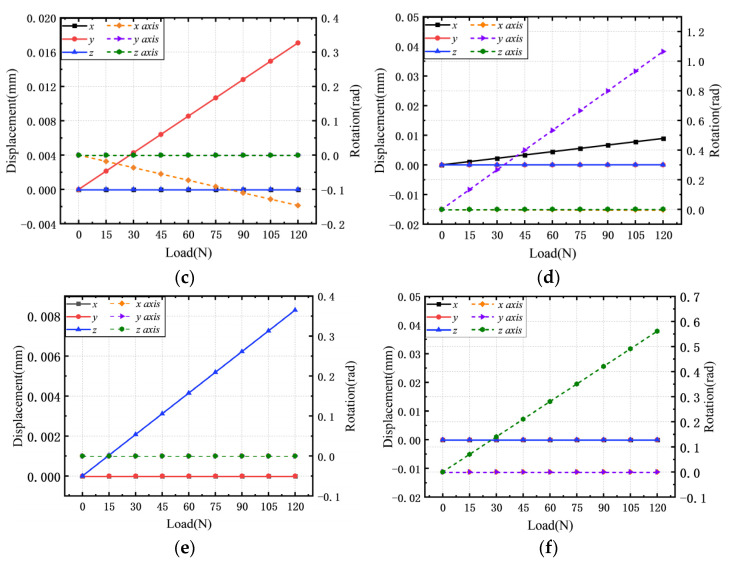
The relationship between input and output from the isogeometric model (solid lines represent the displacements and dashed lines express the rotations). (**a**) The displacements and rotations of each direction when loading in *F_x_* direction. (**b**) The displacements and rotations of each direction when loading in *M_x_* direction. (**c**) The displacements and rotations of each direction when loading in *F_y_* direction. (**d**) The displacements and rotations of each direction when loading in *M_y_* direction. (**e**) The displacements and rotations of each direction when loading in *F_z_* direction. (**f**) The displacements and rotations of each direction when loading in *M_z_* direction.

According to Equation (17), The change law of output with the input force/moment is obtained in Figure 6.

The linear regression equations of each degree of freedom are as follows:

(18) $$\begin{array}{l} u_{x}=1.44\times 10^{-4}\cdot F_{x},\;u_{y}=1.42\times 10^{-4}\cdot F_{y},\;u_{z}=6.92\times 10^{-5}\cdot F_{z}\\ \theta_{x}=8.87\times 10^{-3}\cdot M_{x},\;\theta_{y}=8.88\times 10^{-3}\cdot M_{y},\;\theta_{z}=4.67\times 10^{-3}\cdot M_{z} \end{array}$$

The displacements and rotations in Figure 6 demonstrated that the compliant mechanism has six degrees of freedom. As shown in Figure 6a, when a force is applied on the *X*-axis, an accompanying rotation around the *Y*-axis will be generated. Similarly, when the *Y*-axis force is applied, an accompanying rotation around the *X*-axis will be generated, as shown in Figure 6b. These are a disadvantage of this structure. Although it can achieve six degrees of freedom, it has a certain coupling between different directions.

## 4. Manufacturing and Experiment

**Figure 7:**
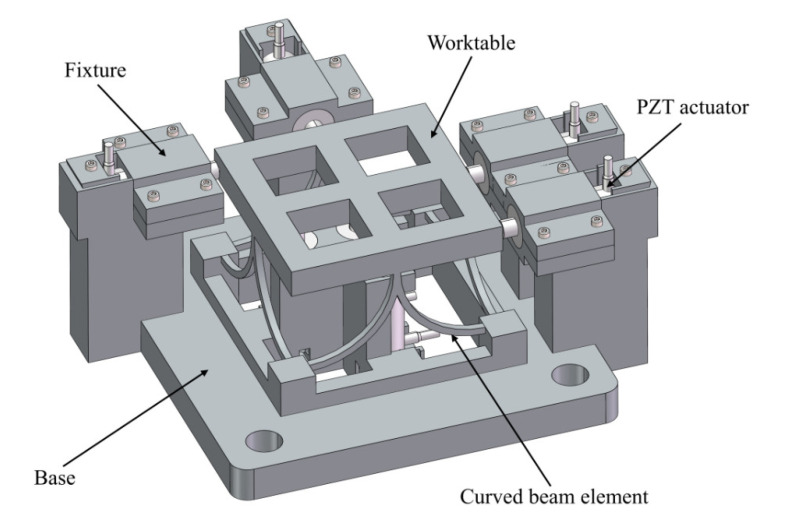
Spatial six degrees of freedom compliant mechanism model for 3D printing.

In order to reduce the error caused by assembly, the compliant mechanism can be manufactured by 3D printing. Then the compliant elements, the worktable, the base, and the fixtures of the actuator are integrated, as shown in Figure 7.

**Figure 8:**
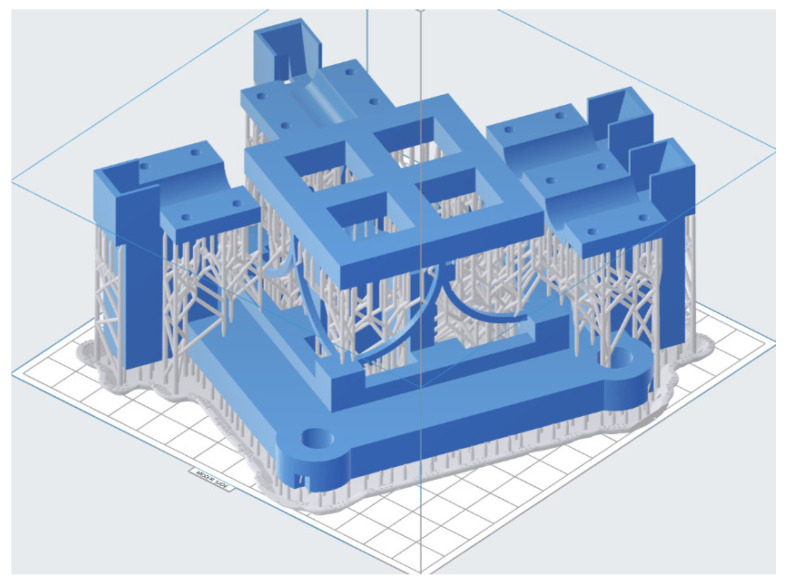
The placement and supports of the compliant mechanism in 3D printing.

The compliant mechanism is manufactured using a Form 3 printer with low-stress light-curing molding technology, and a transparent photosensitive resin material is chosen. The total height of the printed model is 62.75 mm, the layer thickness is 0.1 mm and the accuracy of the 3D printer is 0.025 mm. The model for 3D printing with supports is shown in Figure 8.

**Figure 9:**
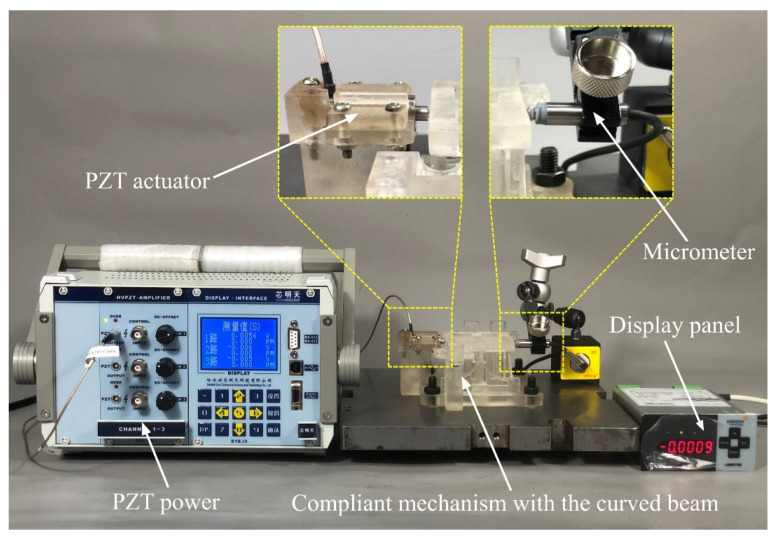
The experimental device with curved beam compliant mechanism and actuators.

The compliant mechanism is driven by piezoelectric ceramic actuators PSt150/7/20 with fast response and nanometer resolution. The driven voltage ranges from 0 V to 150 V, and the displacements are measured by a micrometer. Static driving force should be applied, so the static force loading mode on the control software of the piezoelectric ceramic actuators is selected. The curved beam compliant mechanism and actuators are assembled into an experimental device as shown in Figure 9.

**Figure 10:**
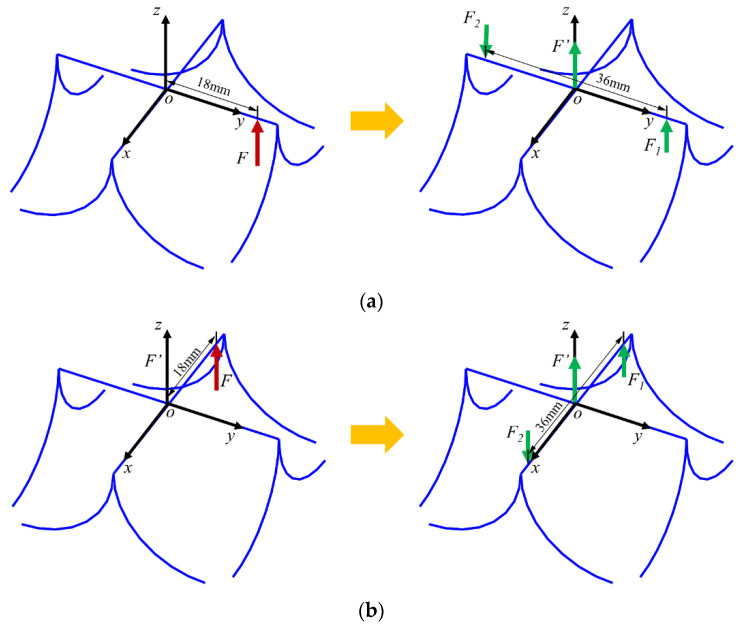
Equivalent relationship between force and moment under *X*-axis and *Y*-axis loading. (**a**) Equivalent relationship between force and moment under *X*-axis loading. (**b**) Equivalent relationship between force and moment under *Y*-axis loading.

Due to the limit of experimental conditions, the rotation is realized by driving a single piezoelectric actuator in the experiment. As shown in Figure 10. The driving load *F* is that of the piezoelectric actuator when rotating along the *X*-axis and its equivalent replacement force is *F′*, *F_1_* and *F_2_*. The rotation of the *X*-axis is realized by driving the load *F* through piezoelectric actuator, and the load *F* can be equivalent to a force *F′* and a moment generated by *F_1_* and *F_2_*. The moment around *Y*-axis is obtained with the same method as the *X*-axis.

**Figure 11:**
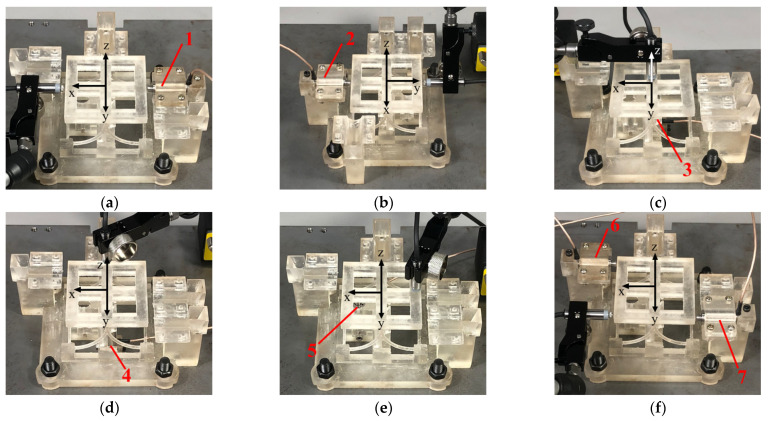
The loading and measuring on six degrees of freedom. (**a**) Loading along *X*-axis direction. (**b**) Loading along *Y*-axis direction. (**c**) Loading along *Z*-axis direction. (**d**) Loading around *X*-axis direction. (**e**) Loading around *Y*-axis direction. (**f**) Loading around *Z*-axis direction.

The displacement along the *X*-axis will be generated if the worktable of the compliant mechanism is driven by actuator 1. Actuator 2 can drive the worktable move along the *Y*-axis. The displacement along the *Z*-axis can be obtained by actuator 3. Rotation around the *X*-axis will be generated if the worktable of compliant mechanism is driven by actuator 4. Rotation around the *Y*-axis can be obtained by actuator 5. Actuator 6 and actuator 7 can drive the worktable rotate around the *Z*-axis. The loading and measuring on the six degrees of freedom of the compliant mechanism are shown in Figure 11.

The input displacements are achieved by controlling the voltage of the piezoelectric ceramic actuators. Under the maximum driving voltage of piezoelectric actuator, the measured displacement along the *X*-axis is 0.0157 mm, the displacement along the *Y*-axis is 0.0158 mm, the displacement along the *Z*-axis is 0.0148 mm, the measured rotation around the *X*-axis is 0.00034 rad, the rotation around the *Y*-axis is 0.000343 rad and the rotation around the *Z*-axis is 0.000182 rad. The displacements and rotations in all directions under the maximum drive voltage are obtained in Table 2.

**Figure 12:**
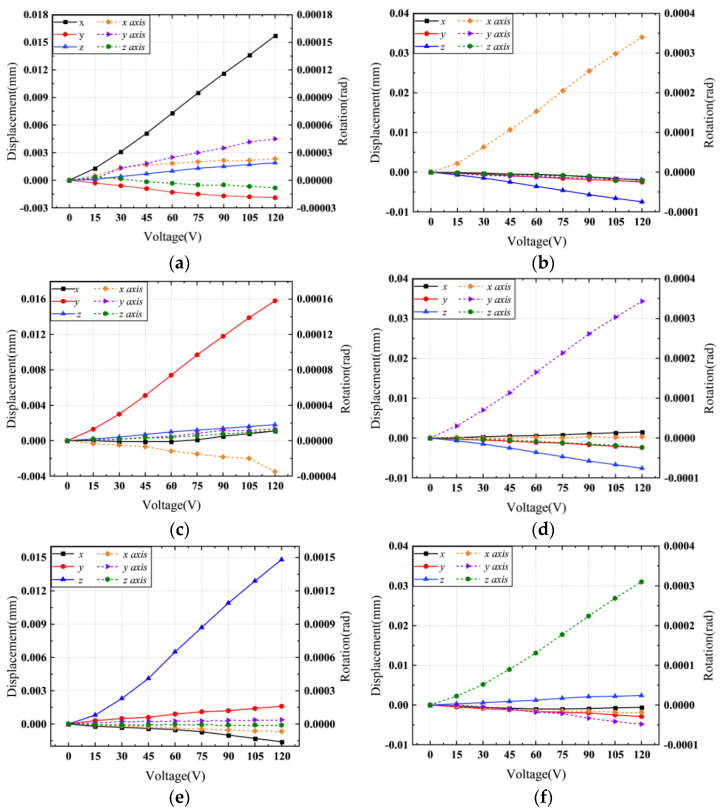
Relationship between the input voltages and the output displacements and rotations in each degree of freedom (solid lines represent the displacements and dashed lines express the rotations). (**a**) Load along *X*-axis direction. (**b**) Load around *X*-axis direction. (**c**) Load along *Y*-axis direction. (**d**) Load around *Y*-axis direction. (**e**) Load along *Z*-axis direction. (**f**) Load around *Z*-axis direction.

Voltages from 15–120 V are applied to the actuators, then the displacements and rotations of each direction are recorded by the micrometer. The relationship between the input voltages and the output displacements and rotations is shown in Figure 12.

The linear regression equations of each degree of freedom direction are as follows:

(19) $$\begin{array}{l} u_{x}=1.35\times 10^{-4}\cdot U-6.1\times 10^{-4},\;u_{y}=1.37\times 10^{-4}\cdot U-6.6\times 10^{-4},\;u_{z}=1.30\times 10^{-4}\cdot U-1.0\times 10^{-3}\\ \theta_{x}=2.97\times 10^{-6}\cdot U-1.8\times 10^{-5},\;\theta_{y}=2.97\times 10^{-6}\cdot U-1.2\times 10^{-5},\;\theta_{z}=2.69\times 10^{-6}\cdot U-1.9\times 10^{-5} \end{array}$$

The slope of the input-output linear regression model obtained by isogeometric analysis and the experiment are shown in Table 3.

From Table 3, we cannot compare whether the theory is consistent with the experiment. The specific reasons are as follows: (1) In the torque loading experiment results, there is only voltage on the *X*-axis and no force arm, because the multiplication of voltage arm has no physical meaning. (2) The dimensions are inconsistent. The input in isogeometric analysis is force or moment, while the input in the experiment is voltage.

For comparison, the data are processed as follows: (1) In the experimental data processing of moment loading, the x coordinate is changed into the multiplication of voltage and force arm. The force arm of the *X*-axis and the *Y*-axis is 36 mm and the force arm of the *Z*-axis is 52 mm. Then the slope is recalculated. (2) Dimensionless processing should be conducted to the slope obtained by isogeometric analysis and the experiment. For the dimensionless process of force, the slope of the *X*-axis loading force and output displacement is used as the benchmark. For the dimensionless process of moment, the slope of the *X*-axis loading force and output displacement is used as the benchmark. The theoretical and experimental slopes obtained after the dimensionless process are shown in Table 4.

According to Table 4, the linear regression equation slope ratios of theory and experiment are closed. Then the correctness of the theoretical model is verified. The difference between the experimental data and isogeometric analysis results may be due to the piezoelectric actuator input error, machining error, assembly error, the gravity of the moving part and the surrounding environmental factors such as temperature. The experimental data demonstrated that the input and output displacements and rotations of compliant mechanisms in the six degrees of freedom have a good linear relation, and the maximum inter-directional coupling rate is less than 12%.

## 5. Conclusions

A simple compliant mechanism with six degrees of freedom based on the curved beam element is proposed. Isogeometric analysis is employed to establish the model of the compliant mechanism. Experiments are conducted to verify the feasibility. The main conclusions are as follows:

Based on the curved beam element, a simple compliant mechanism with six degrees of freedom is synthesized. NURBS is employed to express the curved beam model. The simplified geometric model of the compliant mechanism is established.

The element stiffness matrix suitable for spatial curved beams was derived by isogeometric analysis. The global stiffness matrix of the compliant mechanism was obtained. The static analysis of the compliant mechanism presented the relationship between the input and output.

The compliant mechanism was manufactured by 3D printing. The loading test in six degrees of freedom verified the feasibility of the curved beam compliant mechanism. The relationship between output and input was basically consistent with the isogeometric model. The maximum inter-directional coupling rate was less than 12%.

Although the proposed compliant mechanism solves the contradiction between multi degrees of freedom and a simple structure, the structure has the disadvantage of directional coupling. In the future, we expect to optimize the performance of the compliant mechanism by changing the shape of the curved beam.

## Figures and Tables

**Table 1:** The displacements and rotations under the maximum load of 120 N.

Load	*u*_x_/mm	*u*_y_/mm	*u*_z_/mm	*θ*_x_/Rad	*θ*_y_/Rad	*θ*_z_/Rad
120 N	1.73 ×10^−2^	1.71 ×10^−2^	8.30 ×10^−3^	1.064	1.065	0.561

**Table 2:** The displacements and rotations in all directions under the maximum drive of the piezoelectric actuator.

Driving Direction	*u*_x_/mm	*u*_y_/mm	*u*_z_/mm	*θ*_x_/Rad	*θ*_y_/Rad	*θ*_z_/Rad
*F*_x_	1.57 × 10^−2^	−1.90 × 10^−3^	1.90 × 10^−3^	2.33 × 10^−5^	4.50 × 10^−5^	−8.33 × 10^−6^
*F*_y_	1.10 × 10^−3^	1.58 × 10^−2^	1.80 × 10^−3^	−3.50 × 10^−5^	1.33 × 10^−5^	1.13 × 10^−5^
*F*_z_	−1.60 × 10^−3^	1.60 × 10^−3^	1.48 × 10^−2^	−6.66 × 10^−5^	3.83 × 10^−5^	−1.07 × 10^−5^
*M*_x_	−2.00 × 10^−3^	−2.50 × 10^−3^	−7.50 × 10^−3^	3.40 × 10^−4^	−1.83 × 10^−5^	−2.16 × 10^−5^
*M*_y_	1.50 × 10^−3^	−2.40 × 10^−3^	−7.60 × 10^−3^	3.33 ×10^−6^	3.43 × 10^−4^	−2.33 × 10^−5^
*M*_z_	−6.00 × 10^−4^	−2.90 × 10^−3^	2.40 × 10^−3^	−1.83 × 10^−5^	−4.83 × 10^−5^	3.10 × 10^−4^

**Table 3:** Comparison of slopes after dimensionless process.

Driving Direction	*F*_x_	*F*_y_	*F*_z_	*M*_x_	*M*_y_	*M*_z_
The slope of the isogeometric analysis results	1.44 × 10^−4^	1.42 × 10^−4^	6.92 × 10^−5^	8.87 × 10^−3^	8.88 × 10^−3^	4.67 × 10^−3^
The slope of the experimental data	1.35 × 10^−4^	1.37 × 10^−4^	1.30 × 10^−4^	2.97 × 10^−6^	2.97 × 10^−6^	2.69 × 10^−6^

**Table 4:** The linear regression equation slope ratios between isogeometric analysis results and experimental data.

Driving Force/Moment	*F*_x_	*F*_y_	*F*_z_	*M*_x_	*M*_y_	*M*_z_
The slope from isogeometric analysis after dimensionless process	1	0.986	0.479	1	1.002	0.527
The slope from experimental data after dimensionless process	1	1.017	0.969	1	1.002	0.625
